# How do NIHR peer review panels use bibliometric information to support their decisions?

**DOI:** 10.1007/s11192-017-2417-8

**Published:** 2017-06-12

**Authors:** Salil Gunashekar, Steven Wooding, Susan Guthrie

**Affiliations:** 1 RAND Europe, Cambridge, CB4 1YG UK; 2 Centre for Science and Policy, University of Cambridge, Cambridge, CB2 1QA UK

**Keywords:** Bibliometrics, Peer review, Review panels, Grant funding

## Abstract

Bibliometrics is widely used as an evaluation tool to assist prospective R&D decision-making. In the UK, for example, the National Institute for Health Research (NIHR) has employed bibliometric analysis alongside wider information in several awarding panels for major funding schemes. In this paper, we examine various aspects of the use of bibliometric information by members of these award selection panels, based on interviews with ten panel members from three NIHR panels, alongside analysis of the information provided to those panels. The aim of the work is to determine what influence bibliometrics has on their decision-making, to see which types of bibliometric measures they find more and less useful, and to identify the challenges they have when using these data. We find that panel members broadly support the use of bibliometrics in panel decision-making, and that the data are primarily used in the initial individual assessment of candidates, playing a smaller role in the selection panel meeting. Panel members felt that the most useful measures of performance are normalised citation scores and the number or proportion of papers in the most highly cited *X*% (e.g. 5, 10%) for the field. Panel members expressed concerns around the comparability of bibliometrics between fields, but the discussion suggested this largely represents a lack of understanding of bibliometric techniques, confirming that effective background information is important. Based on the evidence around panel behaviour and concerns, we set out guidance around providing bibliometrics to research funding panels.

## Introduction

Bibliometrics is increasingly used in the assessment of research, both for impact evaluation and for awarding research funding. In the UK, the National Institute for Health Research (NIHR) has employed bibliometric analysis as part of a wider set of information in several awarding panels including the NIHR Senior Investigators, Collaborations for Leadership in Applied Health Research and Care, and Biomedical Research Centres/Units competitions. We discuss the specific details about the use of bibliometrics within these three competitions in the next section. Furthermore, the Research Excellence Framework (REF) exercise in the UK drew on bibliometrics in its assessment of the quality of research produced by UK higher education institutions across all disciplines (REF 2014a). For the REF, universities were asked to submit up to four research outputs (e.g. journal articles, monographs, book chapters, etc.) for each member of staff included in their submissions. These outputs were peer-reviewed by expert sub-panels^1^ in order to assess the quality of the outputs in terms of “originality, significance and rigour.” Some of the sub-panels also used citation information provided by the REF team, which was not intended to be used as a primary tool of assessment but rather as a “positive indicator of the academic significance” to inform the decisions arrived at by the REF sub-panels (REF 2012). The sub-panels that used bibliometric data were primarily those affiliated to the health, physical and life sciences which traditionally have good coverage within bibliometric databases and journal-based outputs.^2^

In Australia, a similar national research assessment exercise is conducted. Excellence in Research for Australia (ERA), the first three rounds of which took place in 2010, 2012 and 2015 aims to identify and evaluate the quality of research at Australian higher education institutes (ERA 2015). Results of citation analyses have been explicitly used as indicators of research quality in the exercise in addition to the peer review of a sample of research outputs, with citation information predominantly used in the science and medical disciplines. Unlike the REF, funding is not directly allocated based on the outcomes of the ERA process.

Given the influence of bibliometrics, it is important to understand how panels use the information provided and how it can best support their decision making. Previous articles have explored the reliability of bibliometrics as an alternative to peer review (Nederhof and van Raan 1993; Aksnes and Taxt 2004). A study commissioned by the Higher Education Funding Council for England (HEFCE) reviewed the role of metrics in research assessment (HEFCE 2015a), finding that there is still some cynicism among the research community around the wider use of metrics to evaluate research (Wilsdon et al. 2015). The report concluded that metrics should not replace peer review but that in some cases, peer-review based decision-making could be complemented with the use of “carefully selected” quantitative indicators (HEFCE 2015b). In this article we explore the use of bibliometrics as a supplementary source of information to inform peer review. The role that bibliometrics plays in decision-making in this context, and the suitability of the metrics provided, are not well understood. Typically, bibliometrics are only one part of the evidence provided to support decision-making by panels, and as such it is difficult to test the impact they make on the conclusions the panels reach.

The literature that examines the use of bibliometric data by selection panels is sparse. One notable exception is a study by Lewison et al. (1999) that looks at the bibliometrics used to inform a panel that selected neuroscience grants. The study examined the results from three surveys, two of selection panel members and one of applicants. The aim was to establish panel members’ and applicants’ knowledge of bibliometrics and to determine which indicators they found to be most useful. The authors found that more than two-thirds of the respondents were in favour of using bibliometrics to inform the decision-making process. With regard to specific bibliometric indicators, the respondents felt that citation-based scores and journal-impact category rankings were the most helpful metrics.

## Context

This article describes the use of bibliometrics by panel members in the selection panels for the following three NIHR Competitions:

1. the *7th NIHR Senior Investigators (SI)* 3 *competition 2013*–*2014* (henceforth referred to as SI 2014);
2. the *NIHR Collaborations for Leadership in Applied Health Research and Care (CLAHRC)* 4 *competition 2013*–*2014* (henceforth referred to as CLAHRCs 2014); and
3. the *NIHR Biomedical Research Centres (BRCs)* 5 */Units (BRUs)* 6 *competition 2011*–*2012* (henceforth referred to as BRCs/BRUs 2012).

The NIHR Senior Investigators are a network of approximately 200 eminent researchers including some of the leaders of clinical and applied health and social care research in England (SI 2017). Senior Investigators are selected through an annual competition based on the recommendations of an independent panel of experts. Ten rounds of the Senior Investigator competition have taken place prior to 2017–2018.

NIHR CLAHRCs are collaborative partnerships between English universities and their adjoining National Health Service (NHS) organisations with the primary goal to carry out world-class, patient-centric research that translates “research findings into improved outcomes for patients” (CLAHRCs 2017). Nine CLAHRCs were established across England in 2008 through an open competition. After a second single stage competition in 2013, 13 new CLAHRCs were launched on 1 January 2014 for a period of 5 years.

Established as the NIHR’s flagship infrastructures, Biomedical Research Centres are large partnerships between NHS provider organisations and universities in England that conduct world-class translational biomedical research across a wide range of themes “to transform scientific breakthroughs into life-saving treatments for patients” (BRC 2017). Similar in terms of structure to BRCs but smaller in terms of size and NIHR funding they receive, Biomedical Research Units were NHS/University partnerships that carried out excellent translational research in specific “priority areas of high disease burden and clinical need”, such as cardiovascular disease, nutrition and dementia (BRU 2016). The first cohorts of NIHR BRCs and BRUs were established in 2007 and 2008, respectively, and following a second, open competition in 2011, an international panel of experts selected 11 new BRCs (BRC 2016) and 20 new BRUs (BRU 2016) that came into existence in April 2012. More recently, following a third, open competition in 2015–2016, funding was awarded to 20 new NIHR BRCs for a period of 5 years from April 2017 (BRC 2017).

In each of these competitions, bibliometric performance of applicants was used as one of the pieces of evidence to support the selection process. As will be highlighted in more detail later on, the types of bibliometric data that were presented to the panels, and the extent to which they were used to inform the selection panels’ decisions varied between the competitions. For example, the guidelines for the CLAHRCs competition guidelines mentioned that the publication lists submitted by applicants “will be subject to an independent bibliometric analysis” and “will be analysed and reviewed to validate both their completeness and relevance to the themes of the proposed NIHR CLAHRC, and relevance to the aims of the NIHR CLAHRC scheme” (CLAHRCs 2013).

Although we have focussed our analysis in this paper on biomedical/health research panels, many of the findings are likely to be more widely applicable to other fields of research.

## Methods

For each of the three NIHR-commissioned competitions, we looked at the selection criteria used and how bibliometric information was intended to contribute to those criteria. This information was obtained from publicly available documents or was provided to us by NIHR. We also looked at the range of bibliometric measures provided to each panel and the format in which it was presented.^7^ We conducted semi-structured interviews with 10 individuals across the three selection panels as follows:

- SI 2014: 4 panel members
- CLAHRCs 2014: 3 panel members
- BRCs/BRUs 2012: 3 panel members

This included the chair of each panel with other interviewees randomly selected. The main objective of the interviews was to establish how panel members use bibliometric data and what influence it has on their decision-making. We also wanted to see which bibliometric measures they find most useful and to identify the challenges they have in terms of using the data. The semi-structured interview protocol is provided in the “Appendix”. We used a semi-structured interview approach as this allowed us to explore different issues with different panel members depending on their areas of interest and knowledge, and reflecting their different levels of expertise with bibliometrics. This approach necessarily means that not all respondents may have provided the same level of input on all areas, and indeed some questions may not have been used with some respondents where they were not appropriate.

Interviews were conducted by telephone and took approximately 1 h. Interviews were recorded with the interviewees’ permission, and the recordings were destroyed after review by the team for the purposes of analysis. Interview information was analysed thematically to extract common information across the interviews, collectively and by competition. Data were collated into an Excel spreadsheet question by question, and common topics identified across the interviews.

After 10 interviews we found that we were approaching saturation—that is, most information had been provided by more than one respondent, and new respondents were not yielding significant new or contradictory information, this suggests the number of interviews was sufficient to give a reasonable impression of the breadth of viewpoints amongst panel members. Our interviews only covered members of NIHR selection panels, so care should be taken when extrapolating our findings to other contexts. However, given the similarity of many funding competitions, we believe that this evidence could provide useful insights in other circumstances.

## Results

### What information do the panels receive?

Table 1 lists the various selection criteria that were used in the selection process for each of the competitions (the bibliometrics-related measures have been italicised).

**Table 1:** Selection criteria used in the three NIHR competitions^a^ (the bibliometrics-related criteria have been highlighted in italics)

SI 2014	CLAHRCs 2014	BRCs/BRUs 2012
1. *High quality and volume of internationally excellent research* 2. Relevant research portfolio to the health of patients and the public 3. High impact of the research on improvements in healthcare and public health 4. High impact of the leadership of the individual on clinical and applied patient and public research. Evidence of contribution to NIHR 5. Strong track record in training and developing researchers including evidence of helping to shape training agendas at regional and national level 6. High involvement of patients and public in the design, execution and implementation of research 7. Evidence of engagement of health planners and policy makers	1. *The quality of the collaboration’s existing applied health research and particularly research targeted at chronic disease and public health interventions* 2. The strength of the track record of collaborative working between the University(ies), NHS organisations, providers of NHS services, local authorities, local commissioners, the life science industry, other NIHR-funded infrastructure, AHSNs and patients and the public that comprise the collaboration 3. The strength of the strategic plan for the NIHR CLAHRCs, clearly describing how it will add value through a step change in the way that applied health research is carried out and research evidence is implemented 4. The existing research capacity and plans for developing capacity for research and implementation of research findings for the benefit of patients and the public 5. The strength of the planned programme of high-quality applied health research to be carried out focused on the needs of patients and improved patient outcomes 6. The clarity and strength of the proposals for activities to facilitate the implementation of research findings 7. The relevance of the research and implementation portfolio to the health of patients and the public 8. Value for money	1. *The quality, volume and breadth of internationally*-*excellent biomedical and experimental medicine research and researchers* 2. Existing research capacity, and plans for increasing capacity including training 3. The strength of the forward strategic plan and ability to generate a step-change in capacity to undertake experimental research in the relevant priority area 4. The relevance of the research portfolio to the health of patients and the public 5. The track record in translating advances in basic biomedical research into clinical research, and pulling through basic biomedical research findings into and benefits for patients, the public and the NHS 6. The strength of the strategic partnerships, including those with industry and other NIHR-funded research Infrastructure 7. Value for money

Note that criteria 1 was not included in the published selection criteria for SI 2014, but was included in the guidance to panel members

Table 2 lists the primary bibliometric indicators that were presented to the selection panel for each competition. Although the majority of the bibliometric indicators were common across the competitions, the presentation of results and nomenclature varied depending on the requirements of the respective competition guidelines.

**Table 2:** Bibliometric indicators presented to the selection panels in the three NIHR-commissioned competitions being examined in this study (SI 2014, CLAHRCs 2014 and BRCs/BRUs 2012)

Bibliometric indicator	SI 2014	CLAHRCs 2014	BRCs/BRUs 2012
Volume (e.g. number of submitted publications, number of publications that could be analysed)	√	√	√
Normalised publication citation impact (e.g. ‘Mean Normalised Citation Score or MNCS; ‘Average of Relative Citations’ or ARC)	√	√	√
Normalised journal citation impact (e.g. ‘Mean Normalised Journal Score’ or MNJS; ‘Average of Relative Impact Factor’ or ARIF)	–	√	√
Number or proportion of ‘Highly Cited Publications’ (HCPs)	√	√	√
Ranks associated with some or all of the above indicators of impact	√	√	√
Presence of the applicant in the top *X*% of the applicant pool (e.g. top 5%, top 10%, 1st quartile, etc.) based on their bibliometric indicator ranks	√	√	–
‘Appliedness’ indicators to provide a ‘proxy’ measure of the level of application of the research	–	√	–
Research output and citation impact by bibliometric field for each applicant	–	√	√
List of applicants that merit ‘special attention’ from the selection panel and the reasons for this	√	–	–

When a particular bibliometric indicator was not presented to the selection panel to inform their judgement (e.g. it was not required as part of the competition), it is represented by a dash in the table

Brief descriptions of the key bibliometric data are provided below:

- **Volume** refers to the number of publications for an applicant within a specified period of time.^8^ This could relate to an individual researcher as in the case of the SI 2014 competition or a group of researchers belonging to collaborative partnership between universities and NHS organisations as in the case of BRCs/BRUs and CLAHRCs.
- Often used a proxy for ‘quality’, the **normalised citation impact** is a measure of an applicant’s publication portfolio based on citation counts. The number of citations is normalised to account for different citation patterns across different subject areas and for differences in the age of papers (and also sometimes to account for different document types), and averaged across the portfolio. This indicator is also referred to as the Mean Normalised Citation Score (MNCS) or the Average of Relative Citations (ARC).
- **Highly Cited Publications** (HCPs) is another citation-based indicator that measures an applicant’s research excellence based on the identification of bibliometrically ‘top-performing’ papers. It refers to the percentage of an applicant’s publications that rank among the top *X*% most cited publications worldwide (the choice of the percentage is arbitrary; e.g. it could be 1, 5, 10% and so on). Like the normalised citation score, the HCP indicator is another proxy for ‘quality’ and is also normalised for year of publication and subject area.
- The **normalised journal impact** is an indirect measure of the expected research impact based on the impact factors of journals in which entities publish their papers. The journal impact ‘score’ is normalised to account for the different citation patterns across subject areas as well as to correct for differences due to the age of publications. This indicator is sometimes used as a proxy for level of ‘ambition’ and is also referred to as the Mean Normalised Journal Score (MNJS) or the Average of Relative Impact Factors (ARIF).
- As CLAHRCs are supposed to focus on applied health research (CLAHRCs 2017), specific to the aims and terms of the 2013 CLAHRC scheme funding call, the selection panel was presented with novel ‘**appliedness**’ **indicators** in addition to ‘standard’ bibiliometric data (such as those listed above). The aim was to provide a proxy measure of the level of application of the research of each applicant.^9^ For example, the Cochrane “Appliedness” Indicator represented the average number of citations received from the Cochrane Database of Systematic Reviews (Cochrane Library 2017), which is an internationally recognised database for health guidelines and policy making.
- In the Senior Investigators competition, ‘**special attention**’ applicants were flagged for the panel (and the reasons for warranting special attention) because the bibliometric ‘scores’ for these applicants were potentially unreliable. For example, these could be applicants who have relatively low coverage in the bibliometric database.

The format of the information provided also differed between the three panels as set out in Table 3. In advance of the SI 2014 selection meeting, the panel was provided with a detailed technical report (approximately 100 pages) containing the findings of the bibliometric analysis of the publications of individual researchers who had applied for NIHR Senior Investigator status. In addition to the individual bibliometric profiles of each applicant, the report described the various aspects of the data and sources, and highlighted the bibliometrically ‘top-performing’ applicants as well as those applicants that warranted ‘special attention’ from the panel.^10^ For the CLAHRCs 2014 and BRCs/BRUs 2012 competitions, the panels were provided with a detailed slide set highlighting the results of the bibliometric analysis undertaken for each applicant. The slide sets were accompanied by short memos summarising guidance on how to interpret the bibliometric analysis. The various caveats and weaknesses associated with bibliometric analysis were explained in the information provided to all three panels (for example, that bibliometric analysis should be used to challenge and inform the selection panel’s decision-making process but should not be used on its own to arrive at decisions).

**Table 3:** Format of the bibliometric information presented to the selection panels in the three NIHR-commissioned competitions (SI 2014, CLAHRCs 2014 and BRCs/BRUs 2012)

SI 2014	CLAHRCs 2014	BRCs/BRUs 2012
Detailed report	Detailed slide set + short memo	Detailed slide set + short memo
Presentation at panel meeting	Presentation at panel meeting	Presentation at panel meeting

## What do the panel members say about using bibliometric information?

In this section, we report on the perceptions of panel members on using bibliometrics in selection panel settings, drawing on a cross-section of experts (*n* = 10) across the three NIHR-commissioned panels (i.e. SI 2014, CLAHRCs 2014 and BRCs/BRUs 2012). We focus on the following 8 key areas, each of which is discussed in turn:

1. What is the level of understanding of bibliometrics within the panels?
2. How is the bibliometric information used by the individual?
3. How is the bibliometric information used in the panel setting?
4. What are the panel members’ views on the specific measures provided?
5. What are the panel members’ views of the format of the information provided?
6. What are the concerns the panel members have about the use of bibliometrics?
7. How important are the bibliometrics to the panels’ decision-making?
8. What other information around publications would panel members like to see?

### What is the level of understanding of bibliometrics within the panels?

*I am not at all an expert in bibliometrics; I just have a general idea of what it is.*

Half of the interviewees reported previously sitting on selection panels that used bibliometrics (some of these were in panel settings beyond NIHR). One panel member noted that the experience gained through selection panels over a number of years, had improved their understanding of bibliometrics data and consequently they were able to “make better use of it” during the selection process. The remaining interviewees had never encountered the use of bibliometrics on selection panels prior to being involved with these competitions. These panel members described their understanding of bibliometrics as “rudimentary”, “cursory”, and “limited”. These members recognised that, at best, their understanding of bibliometrics was at a basic level, and certainly not at the detailed statistical level. Some of them were unsure about the details of the normalisation procedure and the comparability of applicants across different research fields.^11^ Overall, levels of expertise varied considerably, so some form of introduction or briefing is required to make sure that the information is accessible and useful to all panel members.

### How is the bibliometric information used by the individual?

*I rely more on judgement rather than bibliometrics indicators. Bibliometrics is a starting point that would make me look at the papers to make me try and see what I can glean from them and is not the determining factor for me …*

*I certainly use the bibliometrics – it is a significant part – would guess it is somewhere between 10 and 20% of the determinant.*

The majority of the interviewees felt that the bibliometrics analysis was useful to have during the assessment period. Three of the interviewees used the bibliometric ‘rankings’ to help determine their own overall rankings when assessing applications. Another interviewee equated the bibliometrics to a factor like grant support, i.e. it was a measure of the success of a researcher’s or team’s operation. This panel member tended to use grant information to gauge what researchers were doing currently, whereas bibliometrics provided a measure of what had been done in the past.

Many of the interviewees acknowledged that bibliometrics was only one aspect of the decision-making process and that they relied more on their judgement. One interviewee, for instance, used the bibliometrics as a “starting point” or a “sorting mechanism” more than anything else. Another panel member described using themselves as a “template” when assessing applications asking “is the applicant ‘better’ or ‘worse’ than I am?” Two interviewees were also more interested in the journals that the applicants were publishing in rather than “what the computer said about the bibliometric performance.”

Two panel members acknowledged that bibliometrics was not a dominant aspect of their assessment but that it was useful to have access to the data. For example, they would pay extra attention to the bibliometrics for ‘outliers’; or if an applicant performed exceedingly well on one or more of the other assessment criteria but not so well on the bibliometrics (i.e. where there were contradictions between the quantitative and qualitative assessments).

Overall, bibliometrics was used in a variety of ways, from an initial heuristic or a starting point for producing rankings, to a tool for looking at ‘outliers’. No one said that they did not like having the bibliometric information available, though some said that they didn’t use it extensively.

### How is the bibliometric information used in the panel setting?

*My sense is that the bibliometrics are probably at their most influential when the assessors are doing their assessments in their own time. At the actual meeting, the discussion doesn’t focus on the bibliometrics in a great deal of detail.*

*The bibliometrics was an ingredient, part of the sauce rather than the meat on the plate.*

Most interviewees felt that the bibliometric analyses played a greater role during the assessment phase when panel members were carrying out their individual evaluations. By the time the panel met, individual assessors had already taken the bibliometrics information into account in order to “make up their minds.” At the meeting, the discussion did not focus on the bibliometrics in a great deal of detail. As one panel member remarked, “by the time everyone got into the room, the bibliometric rankings had already achieved what [I supposed they were meant] to do in helping reviewers ‘shape’ an overall rank. I didn’t hear a lot of discussion come up about the rankings at that point – they had already done their job.”

The bibliometrics often served as the starting point for discussions. Sometimes, clear reference would be made to an applicant’s impressive bibliometric performance, or the converse. Bibliometrics might also surface at the meeting when presentations by the applicants^12^ were a ‘disaster’ in which case the panel would look much more closely at the bibliometrics to verify whether the applicants were “just having an off day.” Overall, however, the panel discussion centred on the other more qualitative aspects of the evaluation criteria and applicants’ contributions to the field of medical research (such as demonstration of leadership within the research community, helping build capacity and relevance to patients and the public). One panel member commented that “the publications are the ‘scaffolding’ – they are like a structure but you couldn’t possibly do much beyond it unless you have other things.”

Some interviewees pointed out that generally there was no disagreement between panel members regarding the bibliometrics results. In contrast to some panel members’ views regarding the use of bibliometrics data during the assessment period, three interviewees recounted that the bibliometrics data were not used in the discussions about the ‘outlier’ applicants. One panel member noted as applicants increased in quality there was now a greater degree of competition for the limited number of awards. This was where the bibliometrics-related discussions at the panel meetings could come to the fore. One interviewee noted that this was a particularly valuable contribution of the bibliometrics—helping the panel ‘winnowing down’ to the most competitive applicants who they could then concentrate their attention on. Underlining this point, an interviewee commented that “when we get to the tough apples-oranges stuff, it tends to be a lot more reliant on the same old subjective questions” (e.g. contribution to national training agenda; contribution to the day-to-day activities of the NIHR; quality of patient and public engagement work; influence in shaping policy and the work of the NHS; etc.). Another interviewee had more concerns noting there had been instances when he thought the panel relied too heavily on the bibliometrics, particularly when applicants in the “middle” were being discussed. This primarily took place at the end of the meeting, “when you’re getting tired, [and using the bibliometrics] is the slightly ‘lazy option’.”

Overall, there are differing views of how bibliometrics is used at the panel meeting. There is some suggestion that it can be used as an initial filter, and to support decision making about candidates around the funding line in terms of performance. Concern was also raised about potential overreliance on bibliometrics in some cases.

### What are panel members’ views on the specific measures provided?

*For competitions where one is really looking at knowledge translation downstream, having an indicator for research “appliedness” would be really useful.*

In all three competitions, the selection panel was presented with a number of bibliometric indicators for each applicant. The interviewees were asked how helpful they found these indicators in informing their judgement on the scientific track record of applicants.

- *Number of publications* Opinion was split on this indicator. Six interviewees felt that this was a useful or moderately useful bibliometric indicator. One interviewee remarked that while they were used to receiving applications from prolific researchers with a considerable number of publications, they were also aware of the possibility of some applicants trying to ‘game’ the system by not disclosing their ‘weaker’ publications. Another panel member suggested that although volume of scientific production was an important criterion, it would be more helpful to the panel if the volume was nuanced into categories such as academic impact papers, clinical impact papers, policy impact papers, and so on. Three panel members did not think that volume was a useful bibliometric indicator.
- *Average number of citations, normalised for field* 13 All the panel members interviewed found this bibliometric measure to be useful or moderately useful, and more helpful than the volume of publications. As one panel member remarked, this indicator “gives you more confidence of the impact of the work rather than simply relying on the number of publications.” However, concern was raised by one interviewee around how the normalisation (by field/sub-field) was being carried out. This panel member advised that it would be helpful if the panel knew the fields used in the normalisation process.
- *Number or fraction of highly cited publications (HCPs)* This indicator was considered to be useful or moderately useful by all the interviewees. Two interviewees were more persuaded by the HCP indicator than average citation indicators feeling that it “helped to earmark the really good researchers”. For both of these types of measures three interviewees remarked that they would like more details about the fields being used in the normalisation process.
- *Measures related to journals* 14 Opinions were mixed on these measures. Four panel members found them to be useful. One interviewee noted that papers in *Science* or *Nature* “jinx[ed applicants] in a positive way”, however, in general, there was a movement away from the attitude of “just because it is in Nature, it’s got to be right!” Although journal-based bibliometric indicators are not presented to the Senior Investigator panel, one SI panel member felt strongly that they should be. They remarked that it was a “knee-jerk” reaction to look at the journals that the “high end” applicants are publishing in and went on to say that “if you are getting papers in the top journals, then […] I would like to know; whatever your contribution and however you got there, it puts you into a category of great interest.” Two interviewees felt the previous three bibliometric indicators were sufficient (i.e. volume, average number of citations and number of highly cited publications). One pointed out that having a journal based metric would be potentially damaging—it would enhance those researchers who are publishing in the top ranked journals and “it will do nothing to help the people who are publishing good work that penetrates deep into professional practice.”
- *Ranks based on some of the above indicators* Opinions were mixed, with five interviewees suggesting this was a useful indicator and three that this was not particularly helpful.^15^ One respondent remarked that having the rankings was helpful, “particularly when you’re stuck with 4–5 applications [at the funding line] that [in other respects] look quite good” and that it was “a useful way of distinguishing between applicants; it helps knock people off the fence.”
- *Measures of application* Overall, panel members were interested in this type of measure in the abstract, but were sceptical of the ability of bibliometrics to deliver this type of information. Two of the three CLAHRC panel members interviewed were broadly positive about the inclusion of novel ‘appliedness’ indicators to aid the selection process. The third disagreed, explaining that they had relied on their qualitative assessment of ‘appliedness’ and suggesting that the quantitative measures were not particularly helpful although they acknowledge that it was a “noble attempt at an impossible job.” One of the CLAHRC panel members remarked that they felt more confident in the ‘appliedness’ measure when it correlated with one of the primary bibliometric indicators of citation impact. Even though measures of application were not provided to the SI 2014 and BRCs/BRUs 2012 competitions, the respective panel members’ views were sought on this potential indicator. Three SI 2014 panel members and one BRCs/BRUs 2012 panel member expressed interest in such measures. One of the SI 2014 panel members questioned whether the ‘appliedness’ measures could be used to highlight research in highly-specialised, “implementation-type” journals that traditionally might have been overlooked.

### What are the panel members’ views of the format of the information provided?

*The bibliometrics results and analysis were laid out in a clear and reasonable manner.*

*I haven’t been on any other panels that do this – other panels just give you numbers and you’re on your own. I like the fact that the panel gets to talk to the experts and that we have the chance to ask some questions about the bibliometric data… It is perhaps one of the most useful steps… and is unique to NIHR panels.*

For the SI 2014 competition, a detailed technical report containing the results of the bibliometric analysis was submitted to the panel approximately 3 months in advance of the panel meeting. For the BRCs/BRUs 2012 and CLAHRCs 2014 competitions, a detailed slide set and a 3–4 page summary memo was sent to the panel in advance of the meeting. Some panel members commented that they read the material “cover to cover”, however, they were not sure whether this applied to other panel members. Most panel members thought the materials provided were useful and assisted them during their assessment of the applicants. None of the panel members recommended major changes to the bibliometrics materials, noting that they were “just about right.” One interviewee on the SI 2014 panel noted that they would have found a shorter summary slide set useful and that it would have been useful to have received it even earlier in the assessment phase.

**Fig. 1:**
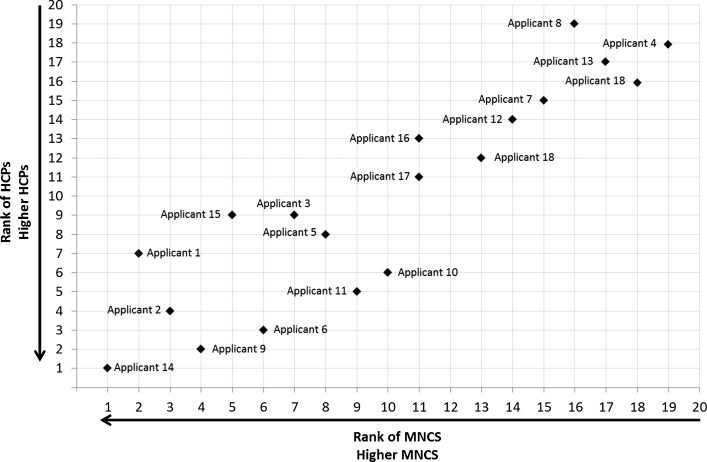
Dummy example of a typical scatter plot showing data on the rank by Mean Normalised Citation Score (MNCS) and rank looking at Highly Cited Publications (HCPs) graphically for a number of applicants. Similar scatter plots were used to present data to all three selection panels

A number of the interviewees said they liked the way potentially complex data had been presented in the report/slides, in particular, the use of scatter plots, tables, and sorted lists. A dummy example of a typical scatter plot of the rank of the number of highly cited publications versus the rank of the mean normalised citation score is shown in Fig. 1. Three panel members especially noted the value of having the list of ‘special attention’ applicants at hand when assessing applications. Panel members appreciated the opportunity to ask questions about the data during the assessment.

Most interviewees found it was helpful to have the key results from the bibliometric analyses presented at the start of the panel meeting. One panel member who had previously been on several non-NIHR panels said that this was the first time they had been given the chance to interact with the research team that carried out the analyses. Most of the interviewees noted that the length of the presentation (15–20 min) was appropriate and they were “pleased” to be reminded of the analyses and to be able to have the opportunity to raise any questions. Some interviewees suggested that the presentation was used solely for clarification purposes (e.g. about the methodology, indicators, normalisation, etc.), and that it did not affect the eventual judgements made by the panel. However, one interviewee pointed out that the discussion which followed the presentation was “asymmetrically intense and long” relative to how the bibliometric data are used by the panel. Therefore, as noted by one of the panel members, it was important that they “kept cracking on” during the meeting to avoid a disproportionate amount of time being spent discussing the minutiae of the analysis. It should also be noted that given panel members indicated that the bibliometric information is primarily used at the individual assessment stage, it is important that adequate information is provided in advance of the meeting rather than relying on the presentation at the panel meeting to explain important issues or caveats.

Overall, the panel members broadly found the information provided useful, particularly the use of graphical and tabular formats in the material provided in advance, and the presentation and Q&A session at the start of the panel meeting.

### What are the concerns the panel members have about the use of bibliometrics?

*I have noticed that on the panel, the citation metrics are increasingly advantaging those people who come in with extremely heavy weight publications generally from the sciences type of background (e.g. clinical laboratory type of people). They can generate a larger volume of publications because laboratory experiments take much less time to complete and they can also generate more ‘heavy-hitting’ publications in terms of getting articles published in e.g. Nature, Genetics, etc. It is fundamentally disadvantaging the people who are spending ‘5 years’ collecting data in one clinical trial that may or may not report in the Lancet based on whether or not it gets a positive or negative result. This automatically severely disadvantages applied health researchers in any citation metric.*

*In principle, it’s a very welcome undertaking. Bibliometrics is a very useful basis for doing what is an almost impossible job of comparing very nice apples with very nice oranges. Without the bibliometrics input, the process would take six weeks and come to no better conclusion.*

*I’m in favour of bibliometric information being available to the panel. I would not be in favour of them being incorporated as part of the formal criteria used to assess applications.*

In general, most interviewees were in favour of having bibliometric information to support their decision-making. However, there was a divergence in views around whether bibliometrics should be incorporated as an explicit evaluation criterion in competitions such as Senior Investigators, BRCs/BRUs and CLAHRCs in the future. Three panel members were against the idea of bibliometrics being included as one of the formal assessment criteria. Five panel members acknowledged that bibliometrics was a key element of a thorough assessment process. One interviewee noted that the “the idea of throwing out bibliometrics at this stage is a non-starter” with another commenting that it was very useful to “separate people who are close to the [funding] line.” It is important to note that even the panel members who supported the inclusion of bibliometrics in the assessment process highlighted the importance of the interpretation of the data. Some panel members wanted more sophisticated bibliometric analysis that went beyond “bean counting” and helped illustrate some of the more qualitative aspects of an application.

However there were also concerns that panel members were occasionally “hiding behind the numbers” and putting too much weight on the bibliometric scores, coming up with assessments that “matched the bibliometrics” without making a detailed assessment of each applicant. One panel member suggested that “metrics always drive strange behaviours both of panels and of individuals, but we live in a metricised world.” Another panel member commented that they would be “fascinated to see what would happen if for 1 year, you didn’t provide any bibliometrics to the panel.”

Panel members had some specific concerns about biases that bibliometric measures might introduce:

- *Bias against certain groups of researchers* An interviewee commented that there may be bias against early career and part-time researchers in these competitions since they have less time to accumulate publications (and consequently citations) compared to more established and full-time researchers. Similarly, there was also a concern that asking applicants to submit a minimum number of publications over a given period of time would discriminate against women who took career breaks. A recent bibliometric study by Larivière et al. (2013) confirms that global and disciplinary gender imbalances exist in scientific research. For example, the study finds that on a global scale, female researchers account for less than 30% of fractionalised authorships and there are almost twice as many male first-author articles than there are female. Furthermore, articles published by women in prominent author positions (i.e. first- or last-authorship) tend to attract fewer citations. However, there is evidence that similar gender biases exist in peer review (Bornmann et al. 2007).
- *Bias due to differences between fields* Questions were raised by some of the interviewees over the issue of field normalisation: i.e. does normalising by research field get rid of the apparent bias between basic and applied researchers? Based on the discussion at interviews, it seems likely that most of the scepticism about the field-based normalisation of citation-based indicators of impact is a result of misunderstanding around the process used for field normalisation. As one interviewee observed, “the use of bibliometrics as an evaluation criterion does in fact level the playing field so that everybody involved gets a fair chance of being able to make their contribution.” This suggests there is a role for further/better explanation of the normalisation process. However, concerns may also relate to questions about the effectiveness of the normalisation process, and pertinent questions are being asked in the broader metrics community about the ideal level of aggregation for field normalisation in bibliometrics (Wouters et al. 2015).
- *Bias due to differences between research types* Some interviewees mentioned that during the panel meeting the spectrum of research from upstream, laboratory-based work to downstream, highly applied research is widely discussed, and in particular, how relative ‘values’ are affected by the bibliometrics. For example, some interviewees suggested citation metrics appear to be “working in favour” of those applicants with extremely “heavy-hitting” publications—typically applicants with traditional science backgrounds (e.g. clinical laboratory researchers who publish in journals such as *Nature* and *Genetics*). It was felt that this automatically “severely disadvantages” applied health researchers who could, for instance, spend several years collecting data in a single clinical trial that, depending on the results, may or may not report in one of the ‘top’ journals. Questions were therefore posed around whether there is a reasonable balance between upstream and applied researchers in the outcome of the competitions, and the broader impact this could have on specific fields of research like allied health.

### How important are the bibliometrics to the panels’ decision-making?

*Bibliometrics is right up there but there is also a lot of other stuff to also weigh in.*

*Bibliometrics, grants, etc. are all proxies and we take them! At the end of the day, you actually have to read the proposal.*

Notwithstanding some of the concerns highlighted, bibliometrics were still acknowledged as a significant element of the assessment process. The majority of those interviewed appreciated having the bibliometrics data available to inform their decision-making, but stated that it was not the determining factor, with one exception who felt the bibliometric information had too much influence. It was utilised more as a “validating mechanism” and none of the interviewees explicitly assigned a specific ‘weight’ to the bibliometrics during their assessment.^16^ Furthermore, as indicated earlier, the bibliometrics had a greater influence on panel members’ individual assessments and served predominantly as a starting point for discussions in the panel meetings where final selections were made. Many panel members had the impression that applicants who come out on ‘top’ overall in the evaluations were often those with the strongest bibliometrics performance. Another panel member pointed out that they were more interested in knowing whether an applicant had published 1–2 particularly significant papers that (for example) “changed the way we think about science,” as opposed to numerous “decent” papers containing incremental results and observations. This view was echoed by another interviewee who also asked whether the bibliometrics analyses could be made closer to what the majority of the panel members were looking for, that is, “how do we find out whether the person has done something that is truly impactful?”

### What other information around publications would panel members like to see?

*Give me your top 5 papers so that they can be studied in more detail and more data can be gathered on them… ‘What would you like written on your headstone’ type papers*

Nine of the ten panel members agreed that it would be useful to ask candidates to identify what they considered their “top X” publications. This could be provided as supplementary information to give a more qualitative perspective beyond the bibliometric measures and broadens the way panels assess applications. It was suggested that candidates could also be asked to indicate why they felt these were their ‘best’ or ‘most successful’ publications. One interviewee proposed that the panel could even be requested to do a “deeper dive” on these papers, similar to what occurred in the 2014 Research Excellence Framework in the UK (REF 2014b). Others suggested that closely studying these “all-time great papers” could be made optional for the panel members—for example, they could be given the choice of reading through the abstracts instead, as this would not significantly add to the burden in what was an already long assessment process. One panel member proposed that these publications could be a sample of the most highly cited publications. Alternatively, these papers may not be in the highest impact journals and/or the applicants may not even be one of the principal authors, but their contribution to the paper(s) should have been worthy of note. To some extent this would “do some of the work for the panel because you get a good feel for what the applicants think their greatest achievements are.” Another interviewee suggested that it would be helpful to have citation data on this sample of papers.

## Concluding remarks

In this paper, we have investigated the ways that selection panels employ bibliometric information to support their decision-making processes and inform their judgements. Specifically, the objectives of the work were to determine what influence bibliometrics data has on their decision-making; to see which types of bibliometric measures they find more and less useful; and to identify the challenges they have in terms of using the data. We have examined these issues in this study with the ultimate aim of improving the provision of bibliometric data for the use by panels. The key messages are summarised below.

- The majority of panel members interviewed felt that the bibliometrics analysis, as an assessment tool, was a welcome undertaking. In particular, they felt that the bibliometrics analysis was very useful to have during the individual assessment period. There was strong consensus, however, that the results of the bibliometric analysis should be used in combination with other evaluation criteria (e.g. qualitative peer review and case studies), and not used in isolation for decision-making.
- The results of the bibliometric analyses were more influential during the individual assessment phase when the panel members were carrying out their own evaluations of applications. By the time the panel meeting was reached, assessors had already taken the bibliometrics data into account to make up their minds. The bibliometrics served predominantly as a starting point for discussions in the panel meetings in which the final selections were made.
- The panel members were generally happy with the bibliometric information they received ahead of the panel meetings, and with the opportunity to ask questions about the bibliometric data both during the individual assessment period and at the selection panel meeting. However, given the main role of the bibliometric analysis is at the individual assessment stage, this should not be a substitute for adequate detail and clear presentation in the earlier written material.
- Of all the bibiliometric indicators provided to the panel, highly cited publications and the average number of citations (both indicators normalised for field and age) were considered by far the most useful indicators to make judgements about the scientific quality and impact of applicants. There were mixed views about the use of bibliometric measures related to journal impact, and when it was not provided as part of the bibliometric analyses (e.g. in the Senior Investigator competition), some of the panel members would still use the names of journals that applicants had published in to inform their judgments.
- Because of ‘information overload’, the panel members would not like to be presented with more bibliometric information (e.g. having a detailed slide set instead of a report would be an option). Rather they were more in favour of strengthening and clarifying the interpretation of the existing bibliometric data that was being presented to the panel. Despite these concerns about information overload, there was strong agreement that the panels would like to see the applicant’s own ‘top achievement’ papers flagged in the application form (and potentially the reasons for highlighting these papers as well). The panel members also liked the idea of introducing a REF-style ‘deeper dive’ of the ‘best X’ publications, although some concerns were expressed about the additional burden that this would introduce.
- Panel members had some concerns about potential biases:
  - Some had concerns about the normalisation processes and whether the bibliometric ‘scores’ of applicants across different fields of research could be reliably compared to each other. Based on the comments made at interview, this may to a large extent be due to a lack of understanding of the process. Panel members would like to be presented with more information about this, particularly with regard to the research fields used in the normalisation process.
  - Questions were raised about the relative bibliometric performance of applied versus basic research applicants and the impact this could have, in the long run, on the NIHR landscape.
  - Some expressed concerns about biases surrounding gender and early career researchers in bibliometrics analyses.
- Many of the panel members (and particularly those on the CLAHRCs panel) liked the idea of developing a reliable indicator that could measure the level of research ‘appliedness’ of an applicant’s publication portfolio, but there were concerns over specific indicators currently used in this way.
- A minority of those interviewed were worried that some of the panel members were overly reliant on the bibliometric scores, and no panel members suggested that it was used too little.

## Looking ahead

Based on the data collected during this study as well as our experience of providing and interpreting bibliometric information for previous NIHR competitions, we present the following good practice advice with regard to the use of bibliometric data for selection panels.

- If bibliometrics-related information was to be used as a formal selection criterion (e.g. as a proxy for research ‘impact’ or excellence’), panel members and the academic community more widely would be unlikely to be comfortable with it being used in isolation for decision making. Rather, it should be used complementary to other evaluation criteria (such as qualitative peer review and case studies).
- Do not overwhelm panel members with excessive bibliometric data as the workload of panel members is already relatively high. Panel members typically find normalised citation scores and numbers of highly cited papers the most useful metrics, so focus on these. As some of the data can be relatively complex to interpret, make effective use of visualisations when presenting the data to the panel (e.g. scatter plots and comparative tables).
- Provide the bibliometric results to the selection panel well in advance of the final panel meeting so that the assessors have ample time to interpret the data and inform their individual evaluations.
- Give selection panel members the opportunity to challenge the bibliometric data—both during the individual assessment period as well as during the selection panel meeting by enabling the panel to ‘talk to’ to the bibliometric experts.
- Provide panel members with ‘training’ in the use of bibliometrics to support and inform decision-making.
  - A simple way to do this is to allow bibliometric experts to present the results during the panel meeting, as described above, with the opportunity for panel members to ask questions.^17^
  - Furthermore, along with the bibliometrics data, the panel could be provided with a concise (maximum 2 pages) ‘quick reference guide’ focussing on how to interpret the bibliometric analysis (e.g. explanations of the key bibliometric indicators of impact, fundamental points related to the normalisation process, and the comparability of results across the applicants).
  - In either case, it is important to highlight the caveats and drawbacks associated with bibliometrics analysis when presenting the data to the panel (this may include indicating candidates which you think warrant ‘special attention’ i.e. where the bibliometric measures may be misleading).

## Appendix: Interview protocol

### Background questions

1. What is your understanding of what bibliometrics is and how it can be used for candidate selection?
2. How much experience of using bibliometrics do you have?
3. Was your involvement with the SI 2014/CLAHRCs 2014/BRCs/BRUs 2012 panel the first time you have been on a selection panel that uses bibliometrics as one of the selection criteria?
  - [If not, how long have you been on selection panels that award grants, etc. that use bibliometrics to inform decision-making? Names of previous ‘competitions’ you have been involved with as a member of the selection panel?]

### Utility of bibliometric indicators and challenges

4. How do you use the bibliometric information that is presented to you in your assessment?
5. What influence do the bibliometric data have on your decision-making?
  - How much importance do you attach to the bibliometric element of the selection criteria?
6. You are presented with a number of bibliometric indicators for each applicant; how helpful do you find each of these indicators in informing your judgement on the scientific track record of applicants? [not useful; moderately useful; very useful; not applicable]
  - Number of publications;
  - Average number of citations, normalised for field;
  - Number or fraction of highly cited publications (HCPs) (e.g. in top *X*% of field);
  - *For BRCs/BRUs 2012 and CLAHRCs 2014:* Measures related to the journals in which the publications appear;
  - Ranks based on some of the above indicators;
  - How do you think the indicators should be combined to produce ranks?
7. Which of the above indicators do you focus on in your assessment? Why?
8. What are your concerns or reservations about using the bibliometric data?
  - Normalisation;
  - Coverage—of academic publications;
  - Are there academic types of publication/scholarship that we miss;
  - Are all citations equivalent;
  - Negative citations; and
  - Measures of application.
9. *For SI 2014:* Do you think those concerns are well captured by the special attention measure?^18^
  - Are there better ways this could be addressed?
  - How do you treat special attention candidates?
10. How do you take the various caveats and limitations of bibliometrics (that are presented) into consideration while arriving at decisions?
11. Would you propose that applicants list their “best” 5 publications (for example), so that you have the choice of going through some of these articles during the assessment process?

### Panel meeting

12. During the panel meeting, what elements of the bibliometric analysis do you focus the discussion on?
  - With particular regard to the bibliometric results, how do you go about, as a panel, arriving at a “consensus” decision?
  - How do you, as a panel, deal with “disagreements” with regard to the bibliometric results?
13. If you use “scores” for your assessment, do you moderate these “scores” for bibliometrics after discussion with other panel members?
14. What other criteria, other than bibliometrics, do you use to assess applications?
15. How do you go about “combining” the different elements of the selection criteria?
16. Before the panel meeting, do you approach the Department of Health/NIHR for clarifications regarding the bibliometric data? After the panel meeting, do you approach the Department of Health/NIHR for clarifications regarding the bibliometric data? If so, what are the kinds of clarifications that you seek?

### Improvements

17. Could the bibliometric data be presented to the expert panel in a more effective way? If so, could you suggest ways in which this could be done—have you been on panels where the information has been presented better?
18. Report and/or detailed slide set?
  - Do you find the briefings/presentation of the bibliometric results useful? If so, in what way?
  - If you had a preference, would you like the presentation to be longer or shorter? If longer, what parts of the presentation would you rather not have? If shorter, what additional points would you like to see covered?
  - *For SI 2014*: Do you read the full report (or go through it in detail) before the panel meeting? Which parts of the report are most useful to you? Which parts of the report do you not read at all? Would you just prefer a detailed slide pack instead of a full report?
  - *For CLAHRCs 2014/BRCs/BRUs 2012*: Would you have also liked to have received a detailed ‘report’ in addition to or instead of the slide set that was sent to the panel in advance of the panel meeting?
19. Would you like to see additional or different bibliometric information presented to you in the bibliometric analysis to assist you in arriving at decisions? If so, what are these?

### Concluding question(s)

20. Broadly speaking, are you in favour of having bibliometrics data to inform the selection criterion for awarding major grants, etc.? Yes/no—why?

## Footnotes

1 Specifically, 36 subject-based units of assessment (UOAs) working under 4 main panels.

2 The following REF sub-panels made use of citation data: Clinical Medicine; Public Health, Health Services and Primary Care; Allied Health Professions, Dentistry, Nursing and Pharmacy; Psychology, Psychiatry and Neuroscience; Biological Sciences; Agriculture, Veterinary, and Food Science; Earth Systems and Environmental Sciences; Chemistry; Physics; Computer Science and Informatics; and Economics and Econometrics.

3 See SI (2017) for more information.

4 See CLAHRCs (2017) for more information.

5 See BRC (2016) for more information.

6 See BRU (2016) for more information.

7 Since we (RAND Europe) provided the bibliometric analysis to these panels, we have full access to these data.

8 For example, Senior Investigator applicants were asked to provide information on all their peer-reviewed publications between 1 January 2003 and 31 December 2012. CLAHRC applicant units were asked to submit their top 300 peer reviewed publications published between 1 January 2002 and 31 December 2011. Each shortlisted BRC applicant was asked to submit the top 75 peer reviewed publications by the proposed Director and Theme Leaders, published between 1 January 2002 and 31 December 2010.

9 In addition, a concern was that the bibliometric analysis could be “gamed” by applicants by submitting a higher proportion of basic research publications, which tend to perform better using traditional bibliometric analysis. The indicators designed to measure the “Appliedness” of the candidate units’ research included: (1) Normalised Citations from the Cochrane Database of Systematic Reviews (Cochrane “Appliedness”); (2) Journal Levels from the Patent Board Classification (Journal Level “Appliedness”); (3) Medline Publication Types (Medline “Appliedness”); and (4) the Science-Metrix Composite Measure of “Appliedness”. The Cochrane “Appliedness” Indicator was determined to have the most utility, and was the only indicator included in the main analysis.

10 The SI selection panel is provided with a list of applicants that merit ‘special attention’ and the reasons for being flagged. This list highlights those applicants for whom the computed bibliometric indicators might not be completely reliable (e.g. the bibliometric database coverage may have been particularly low for these applicants).

11 Since the bibliometric indicators of impact are normalised based on the field of publication (and also account for differences in the ages of publications), applicants are not disadvantaged due to differing research practices in different fields. Thus, the bibliometric indicators of impact (e.g. the average number of citations, normalised for field, and the highly cited publications indicator) can be directly compared between applicants from different research fields.

12 In the case of the BRCs/BRUs 2012 competition, partnerships were invited to give short presentations to the selection panel.

13 In the data presented to all three panels, this indicator is normalised to account for different citation patterns between fields (for example, there are more citations in clinical medicine than there are in public health and health services) and to account for variations in the age of publications (since older publications will have the chance to accumulate more citations).

14 For the Senior Investigator competition, bibliometric measures related to journals (e.g. impact factors, normalised impact factors, etc.) were not submitted to the panel as part of the analysis. However, as we have reported in this study, some panel members do tend to look at (in the application materials) the names of the journals that applicants publish into inform their judgement.

15 One panel member did not find the ranking indicators particularly useful because the sample of applications was small enough for them to come up with their own ‘rankings’.

16 One panel member admitted that if they had to “subconsciously put a number on it”, they would assign a 20–30% weight to the bibliometrics element; and that they “wouldn’t give it a zero for sure but also wouldn’t give it a weight as much as 50%”.

17 As noted previously, however, even though we did this (for example, with regard to the SI 2014 competition), some of the panel members still had a few misunderstandings related to the interpretation of the bibliometrics data.
